# Diamond-Structured Photonic Crystals with Graded Air Spheres Radii

**DOI:** 10.3390/ma5050851

**Published:** 2012-05-11

**Authors:** Qingxuan Liang, Dichen Li, Haoxue Han

**Affiliations:** State Key Laboratory of Manufacturing Systems Engineering, Xi’an Jiaotong University, Xi’an 710049, China; E-Mails: dcli@mail.xjtu.edu.cn (D.L.); hanhaoxuesjz@stu.xjtu.edu.cn (H.H.)

**Keywords:** diamond-structured, photonic crystals, stop band, graded

## Abstract

A diamond-structured photonic crystal (PC) with graded air spheres radii was fabricated successfully by stereolithography (SL) and gel-casting process. The graded radii in photonic crystal were formed by uniting different radii in photonic crystals with a uniform radius together along the Г-Х <100> direction. The stop band was observed between 26.1 GHz and 34.3 GHz by reflection and transmission measurements in the direction. The result agreed well with the simulation attained by the Finite Integration Technique (FIT). The stop band width was 8.2 GHz and the resulting gap/midgap ratio was 27.2%, which became respectively 141.4% and 161.9% of the perfect PC. The results indicate that the stop band width of the diamond-structured PC can be expanded by graded air spheres radii along the Г-Х <100> direction, which is beneficial to develop a multi bandpass filter.

## 1. Introduction

Photonic crystals (PCs) are periodic dielectric media in one, two, or three dimensional directions, which form a photonic bandgap and manipulate the propagation of light or electromagnetic waves with a wavelength comparable to the periodicity [1]. Many applications of PCs for various photonic or microwave devices are expected, for such use as waveguides, optical filters, antennas, and sensors [2,3,4,5,6,7]. For some applications, a wider stop band width is favorable so that wave control in a wide range can be realized. The PCs of various structures have been widely investigated through theoretical calculation and experimental evaluation. Among the various structures, diamond structure is one of the most attractive class of structures, which consists of two interpenetrating face centered cubic Bravais lattices [8,9]. It is difficult, however, to form three-dimensional diamond structures because the lattice stacking is difficult to achieve compared with simple face-centered cubic or woodpiles [10,11]. Most of the research efforts on the diamond-structured PCs were concentrated on the PCs fabrication of different operating frequencies [12,13,14,15]. However, little work has been done on broadening the stop band width by graded radii in a PC. Kirihara *et al*. investigated the influence of changing the rod diameter of the lattice separately on the bandgap property in rod-connected diamond-structured PC. In their works, the volume fraction (β) of the dielectric medium was modified from 14% to 33% by changing the rod diameter of the lattice. As the β increased, the bandgap width increased gradually and remained constant near β = 33% [16]. However, diamond-structured PCs with graded air spheres radii have not been studied.

In the present work, a diamond-structured PC with the same lattice constant but a graded air spheres radii was successfully fabricated by stereolithography (SL) and a gel-casting process and its stop band properties along the Г-Х <100> direction were investigated. Experimental results were compared with the Finite Integration Technique (FIT) simulation results.

## 2. Experimental Section

In this research, SL and gel-casting process was employed to fabricate the diamond-structured PCs. Diamond-structured PCs models were designed based on the FIT, and the corresponding PCs molds were fabricated using a stereolithography machine (Product SPS-450B, Hengtong Co. Ltd, Shaanxi, China). This SL system formed a three-dimensional object layer by layer by scanning an ultraviolet laser of 355 nm wavelength over a liquid photopolymer epoxy resin. The diameter of the beam spot was 100 μm with a scanning speed of 90 mm/s in operation and a single layer thickness of 100 μm. The dimensional accuracy of the structure obtained was within 0.1%.

In the gel-casting process, the casting slurry with Al_2_O_3_ powder was prepared firstly. In this process, a premixed solution with a concentration of 25 percent was prepared by dissolving organic monomer (CH_3_CONH_2_, AM) and cross-linking agent (C_7_H_10_N_2_O_2_, MBAM) in appropriate amount of deionized water. After adding an appropriate amount of sodium polyacrylate (25 percent of solid powder by mass) to the premixed solution, Al_2_O_3_ powder was dispersed into the premixed solution progressively. A ceramic slurry with a high solid loading (55 vol %) was prepared after milling for 3 h. After adding initiator ammonium persulfate and catalyst (C_6_H_16_N_2_, TEMED) into the ceramic slurry, the ceramic slurry was stirred in vacuum for 5 min to degas and then smoothly poured into the diamond structure molds. Then the ceramic slurry was polymerized in situ. The parts could be partially unmolded and dried using a freeze drying oven for 36 h under a vacuum degree of 3 Pa (Product DTY-1SL, Vacuum Freeze Dryer, Detianyou Technology co., Beijing, China). Finally, the samples were sintered at 1550 °C for 2 h. The resin prototype was burned out and a ceramic PC of high quality was obtained.

**Figure 1:**
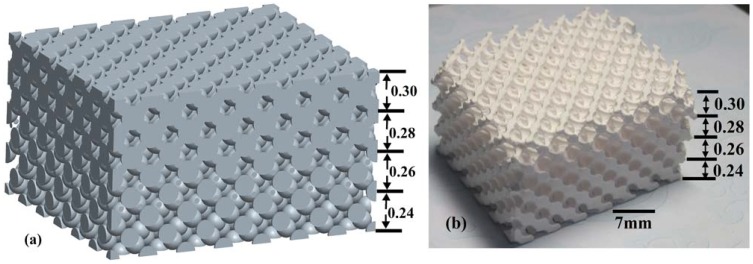
(**a**) The resin mold and the fabricated sample; (**b**) of diamond-structured photonic crystal (PC) with graded air spheres radii.

The lattice constant *a* was fixed at 7 mm, and the stop band was expected to occur in the frequency range of 18–40 GHz. The dimension of the diamond structure was 49 mm × 42 mm × *d* mm, where *d* denoted the length along the Г-Х <100> direction. The radius *r* of the perfect PC was chosen to be 0.30*a*. The radii of air spheres was then decreased gradually by 6.7%, 13.4%, and 20% (the corresponding radius *r* was 0.28*a*, 0.26*a*, 0.24*a*) based on the perfect PC along the Г-Х <100> direction, respectively. First, the perfect PC and three PCs of decreased radii were fabricated. Then the PC with the same lattice constant but the abrupt radii change on the interface between the different photonic crystal regions was also fabricated to investigate its stop band property. The resin mold and the fabricated sample of the PC with graded radii are shown in Figure 1a and Figure 1b respectively.

The transmission amplitude of the PCs was measured in the frequency of 18-40GHz using a free space measurement system with a network analyzer (Agilent E8363B, Agilent Technologies Inc., Palo Alto, CA, USA).

## 3. Results and Discussion

**Figure 2:**
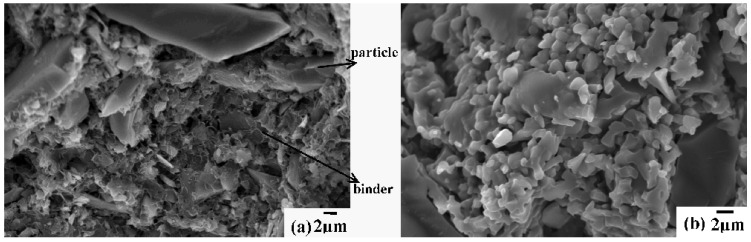
(**a**) The SEM photos of the PC sample after drying; and (**b**) sintered at 1550 °C for 2 h.

Figure 2a shows the SEM photos of the PC samples after drying; Figure 2b shows them sintered at 1550 °С for 2 h. It can be observed, in Figure 2a, that among alumina particles there is a large amount of three-dimensional network organic binder, which provides the green body of the PCs with high strength. In Figure 2b, the organics binder has been burned out, ceramic particles partially went through a phase transition and a porous ceramic structure was formed.

**Figure 3:**
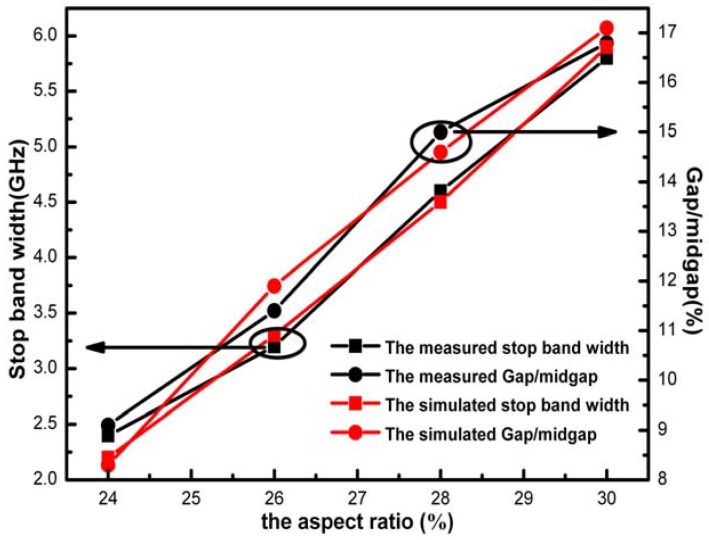
The relationship between the stop band properties and the aspect ratio of the PCs along the Г-Х <100> direction.

Figure 3 shows the relationship between the stop band properties and the aspect ratio of the PCs along Г-Х <100> direction. The dimension of the PCs was 49 mm × 42 mm× 28 mm. In all the cases, the lattice constant α was kept constant and the radius of the air sphere was varied to change the filling ratio. However, the fabrication of diamond-structured PC with air sphere whose aspect ratio is higher than 0.32 or lower than 0.22 is not realistic, so the effective range of aspect ratio is strictly restricted to 0.24 to 0.30. The stop band limits are taken at −10 dB attenuation. It was found that, with the aspect ratio of the PCs increasing, the measured stop band width increased from 2.4 GHz to 5.8 GHz and the gap/midgap ratio increased from 9.1% to 16.8%. Finally the stop band width reaches a maximum value of 5.8 GHz at *r* = 0.30*a* and the gap/midgap ratio shows a value of 16.8%. The simulation result achieved by the FIT was in good agreement with the measurement result. The result indicated that the change of the air sphere radii has an obvious influence on the stop band properties of PC.

**Figure 4:**
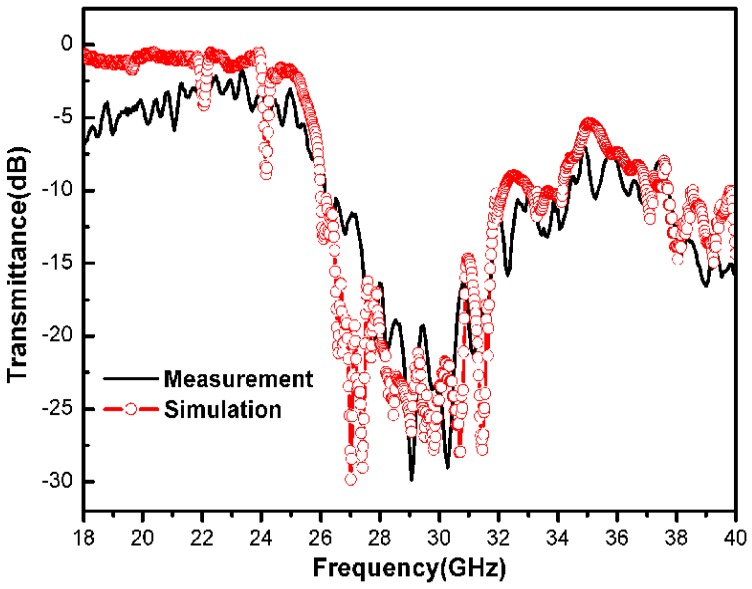
The measurement and simulation result of the PC with graded air spheres radii along the Г-Х <100> direction.

Figure 4 shows the simulation and measurement results of the PC with graded air spheres radii along the Г-Х <100> direction. The dimension of the PC was also 49 mm × 42 mm × 28 mm. It was designed by combining the PC of different radii (0.30*a*, 0.28*a*, 0.26*a*, 0.24*a*) with the dimension of 49 mm × 42 mm × 7 mm along the Г-Х <100> direction respectively. It can be observed that the stop band lies between 26.1 GHz and 34.3 GHz from the measurement result which agreed well with the simulation result attained by the FIT. The stop band width was 8.2 GHz and the resulting gap/midgap ratio was 27.2%, which became 141.4% and 161.9% of the perfect PC respectively. The ultra-wide stop band width is beneficial to develop a multi bandpass filter. The result can be attributed to the graded radii arrangement in the PC which induced the variation of dielectric medium distribution, and thus varies the dielectric contrast effectively. Futhermore, the radii change on the interface between the different PCs regions is abrupt and the stop bands are formed by Bragg scattering. The abrupt radii change on the interface will generate interface scattering waves, which will eventually also strengthen the Bragg scattering and increase the stop band width. The low transmission was also observed from the measurement result due to material absorption induced by the porous ceramic structure.

## 4. Conclusions

Diamond-structured PC with graded air spheres radii was fabricated successfully by SL and gel-casting process and its stop band properties were investigated. It was found that the stop band width was 8.2 GHz and the resulting gap/midgap ratio was 27.2%, which became 141.4% and 161.9% of the perfect PC respectively. The results indicate that the stop band width of the diamond-structured PC can be expanded by the graded air spheres radii along the Г-Х <100> direction, which is beneficial to develop a multi bandpass filter.
